# Characterisation of particles in solution – a perspective on light scattering and comparative technologies

**DOI:** 10.1080/14686996.2018.1517587

**Published:** 2018-10-18

**Authors:** Ciarán Manus Maguire, Matthias Rösslein, Peter Wick, Adriele Prina-Mello

**Affiliations:** a Laboratory for Biological Characterisation of Advanced Materials (LBCAM), Department of Clinical Medicine, Trinity Translational Medicine Institute (TTMI), School of Medicine, Trinity College Dublin, Dublin, Ireland; b AMBER Centre, CRANN Institute, Trinity College Dublin, Dublin, Ireland; c Laboratory for Materials - Biology Interactions, Swiss Federal Laboratories for Materials Research and Testing (Empa), St. Gallen, Switzerland

**Keywords:** Nanoparticles, characterisation, dynamic light scattering, particle tracking analysis, nanoparticle concentration, cause and effect analysis, resonant buoyant mass, resistive pulse sensing, polydispersity, biological samples, 60 New topics / Others, 500 Characterization, 505 Optical / Molecular spectroscopy

## Abstract

We present here a perspective detailing the current state-of-the-art technologies for the characterisation of nanoparticles (NPs) in liquid suspension. We detail the technologies involved and assess their applications in the determination of NP size and concentration. We also investigate the parameters that can influence the results and put forward a cause and effect analysis of the principle factors influencing the measurement of NP size and concentration by NP tracking analysis and dynamic light scattering, to identify areas where uncertainties in the measurement can arise. Also included are technologies capable of characterising NPs in solution, whose measurements are not based on light scattering. It is hoped that the manuscript, with its detailed description of the methodologies involved, will assist scientists in selecting the appropriate technology for characterising their materials and enabling them to comply with regulatory agencies’ demands for accurate and reliable NP size and concentration data.

## Graphical Abstract

**Figure:**
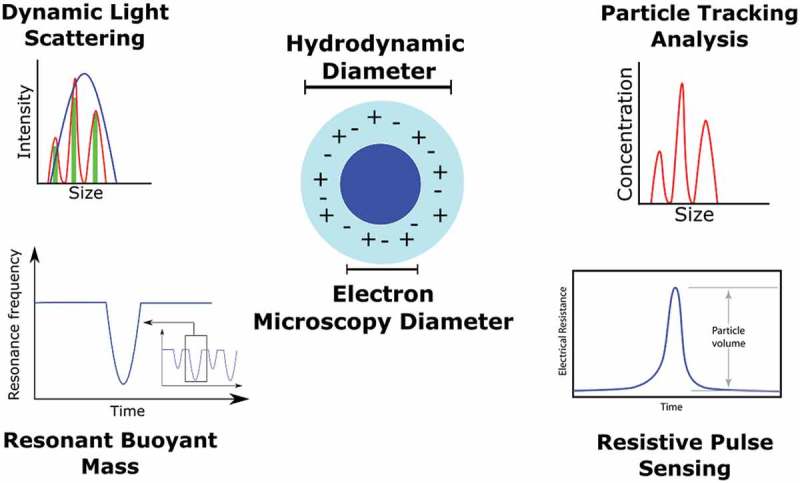

## Background

Accuracy, reliability, reproducibility and robustness are fundamental parameters when it comes to the quality of measurement results and are important factors in the characterisation of nanomaterials. This is particularly true where the physico-chemical properties of nanomaterials give rise to their various consumer and industrial applications. During the production of nanomaterials, characterisation techniques, of an appropriate resolution, are required to monitor and improve the production quality (e.g. batch-to-batch, in-line or online production). Here, the size, along with other physico-chemical properties such as particle concentration or aggregation state, is the most important parameter. As per the European Commission definition, a nanomaterial is such that 50% or more of the particles in a sample have a dimension in the 1–100 nm size range [1]. These same properties may also give rise to unique biological reactivity [2–4], and thus, this has led to mounting concerns over the safety of nanomaterials, and pressure to control the potential risks [5]. To ensure compliance with environmental protection guidelines [6], nanoparticles (NPs) produced, either directly or indirectly, must be fully characterised [7,8]. This has added increasing pressure from industry to ensure compliance with these regulations. Therefore, there is a requirement for sound, statistical data, for a sample that is not influenced by the measurement technique, its inherent uncertainty, or the sample itself, to fulfil these regulatory demands.

Below we set out a description of the principles underlying the most widely used NP sizing techniques, dynamic light scattering (DLS) and particle or nanoparticle tracking analysis (PTA or NTA), their inherent advantages and disadvantages, as well as a cause and effect diagram of the parameters that can influence a measurement by PTA and DLS. Understanding these factors will thus enable researchers to interpret their data in a more meaningful manner, thereby allowing scientists and regulators to make informed decisions that reflect their end uses and applications. We also make reference to other technologies capable of making size and concentration measurements of NPs in solution.

## NP characterisation: different techniques for sizing

Regarding the measurement of the size of a NP, a number of techniques have been developed that operate under various principles. Microscopy techniques, such as transmission electron microscopy (TEM), scanning electron microscopy (SEM), atomic force microscopy, scanning transmission electron microscopy (EM), focused ion beam SEM and helium ion microscopy, can provide information relating to the physical dimension of a NP, but generally do not yield any data about the properties of the NP when it is in solution. In this case, techniques such as Taylor dispersion analysis (TDA), DLS and PTA/NTA can be utilised. This is important to note as the biodistribution, and ultimately biological responses, for example, of the material can be correlated to its dispersion state and solubility [7,9]. To this end, the ability to characterise a nanomaterial sample in solution is of paramount importance where the hydrodynamic diameter is the most widely accepted critical quality attribute. Particle distribution (‘*D*’ value) parameters are also becoming increasingly requested by regulators. *D* values set at 10%, 50% and 90% (*D*10, *D*50 and *D*90) provide valuable statistical distribution insight in the broadness of the particle size range, and emphasise possible potential particle aggregates, within each threshold. These values reflect the diameter of the particles where either 10%, 50% or 90% of the population lies below a certain size. The distribution from PTA/NTA is number based, compared to light scattering intensity for DLS, and as such provides a more ‘true’ reflection of the sample under characterisation.

### Why the hydrodynamic diameter?

The hydrodynamic diameter is the most commonly mentioned particle parameter. It is determined by calculating the Stokes–Einstein equation (Equation 1):

(1) $$D_{t}=\frac{Tk_{B}}{3\pi \eta d}$$

where *T* is the sample temperature, *ƞ* is solvent viscosity, *k_B_* is Boltzmann’s constant, and *d* is the sphere-equivalent hydrodynamic diameter, with the diffusion coefficient (*D_t_*) calculated using varying experimental approaches. It is a crucial parameter when characterising particles as by definition it reflects the size of the particle when in solution and includes coatings or surface modifications made to the particle in question. In contrast, other techniques such as EM typically reflect the ‘dry state’ or internal ‘core’ of a coated or functionalised particle. However, most applications of nanomaterials involve solutions, for example in nanomedicine, and so the hydrodynamic diameter is essential in enabling the correlation of particle sizes with physiological responses. In such applications, the particle surface is in a constantly changing, dynamic state, where proteins in the body absorb onto the particle forming a corona [10]. Changes in ionic strengths and molecules present in the environment can cause changes in the composition and size of this corona, and so the hydrodynamic diameter provides a means to characterise the particle under these conditions [11]. More typical characterisation approaches such as EM imaging would have difficulty in measuring such processes due to the nature of the technique and the possibility of introducing artefacts during sample preparation. Once the particle hydrodynamic diameter is determined, it can then be used to calculate other measures that may be required by the user, such as particles’ volumes and surface areas which may have roles in drug loading or target binding studies.

By definition, the hydrodynamic diameter is the diameter of a hypothetical hard sphere that diffuses with the same speed as the particle being measured. In practice, particles are solvated and can be spherical, spherical-like or non-spherical, while moving dynamically in solution. The determined diameter is therefore an indicator of the apparent size of the solvated particle that is approximated as being spherical. As the particle surface becomes modified, either through functionalisation or agglomeration and aggregation, the approximation of the hydrodynamic diameter changes which can lead to uncertainties in the subsequent calculated dimensions. This can be critical for high-resolution analysis or when adopting highly resolving instruments, where the results can be found questionable. However, by adopting appropriate techniques, reference materials and protocols, then one can obtain very valuable information concerning the particle surface, if the measurements are done correctly, thereby aiding in the characterisation of functionalised materials, for example [12,13].

### Approaches for measuring hydrodynamic diameter: DLS, PTA and NTA methods

**Figure 1.**
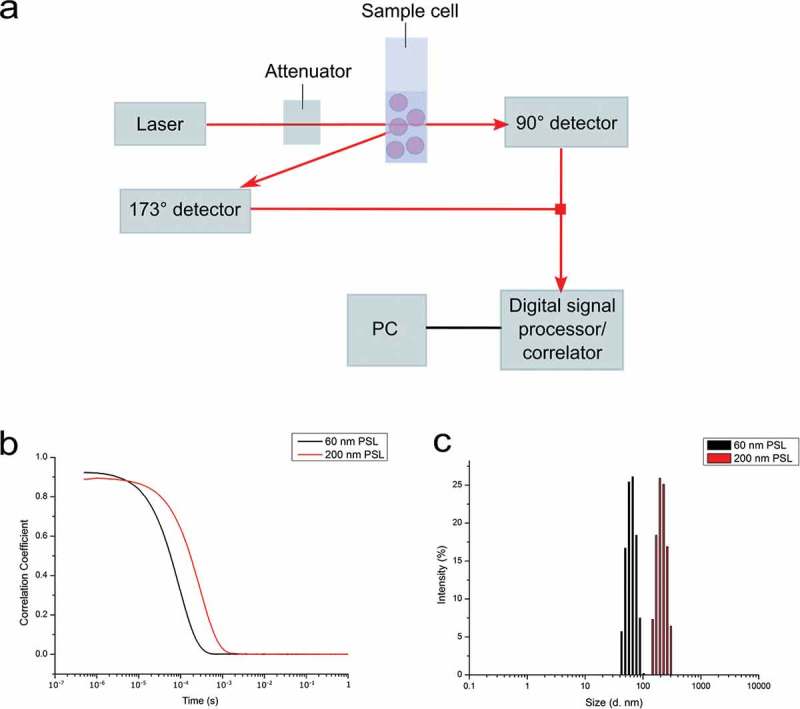
Schematic of a typical DLS set-up. Particles in suspension cause the scattering of light from a laser beam, with detectors (either forward 90°, backward 173°, or multi-angle) recording the fluctuations in the scattered light intensity (a). The fluctuations in scattered light are then used to derive the autocorrelation function, with the decay rate of this curve being proportional to the diffusion coefficient (b). The diffusion coefficient is then used to calculate the particle hydrodynamic diameter (c). Nanomaterials used for DLS measurements are commercial polystyrene latex (PSL) nanoparticles of 60 and 200 nm in diameter.

DLS and more recently PTA and its most common variant, NTA, are becoming conventional techniques for the characterisation of NPs in suspension. They are being developed into standards for the measurement of particle size distributions (PSDs) by the American Society for Testing and Materials (ASTM) [14,15] and International Organization for Standardization (ISO). Both DLS and PTA operate on the principle of light scattering and determine the particle sizes assuming Brownian motion of NPs. However, they differ in the manner in which the data are acquired. DLS measures the fluctuations in scattered light intensity with this being correlated to the particle hydrodynamic diameter via the correlation function and the Stokes–Einstein equation [16]. However, due to Rayleigh theory, the intensity of scattered light is proportional to the sixth power of the diameter, and thus the analysis is heavily weighted towards larger particle size. As a result, NP aggregation will distort the PSD [17]. A schematic representation of a typical DLS set-up is shown in Figure 1. Here, light scattered from the particles is detected, with fluctuation in the scattered light intensity being measured over time. This is then used to generate the autocorrelation function, with the decay in the curve being proportional to the particle diffusion coefficient. The particle hydrodynamic diameter can then be calculated using Equation 1.

**Figure 2.**
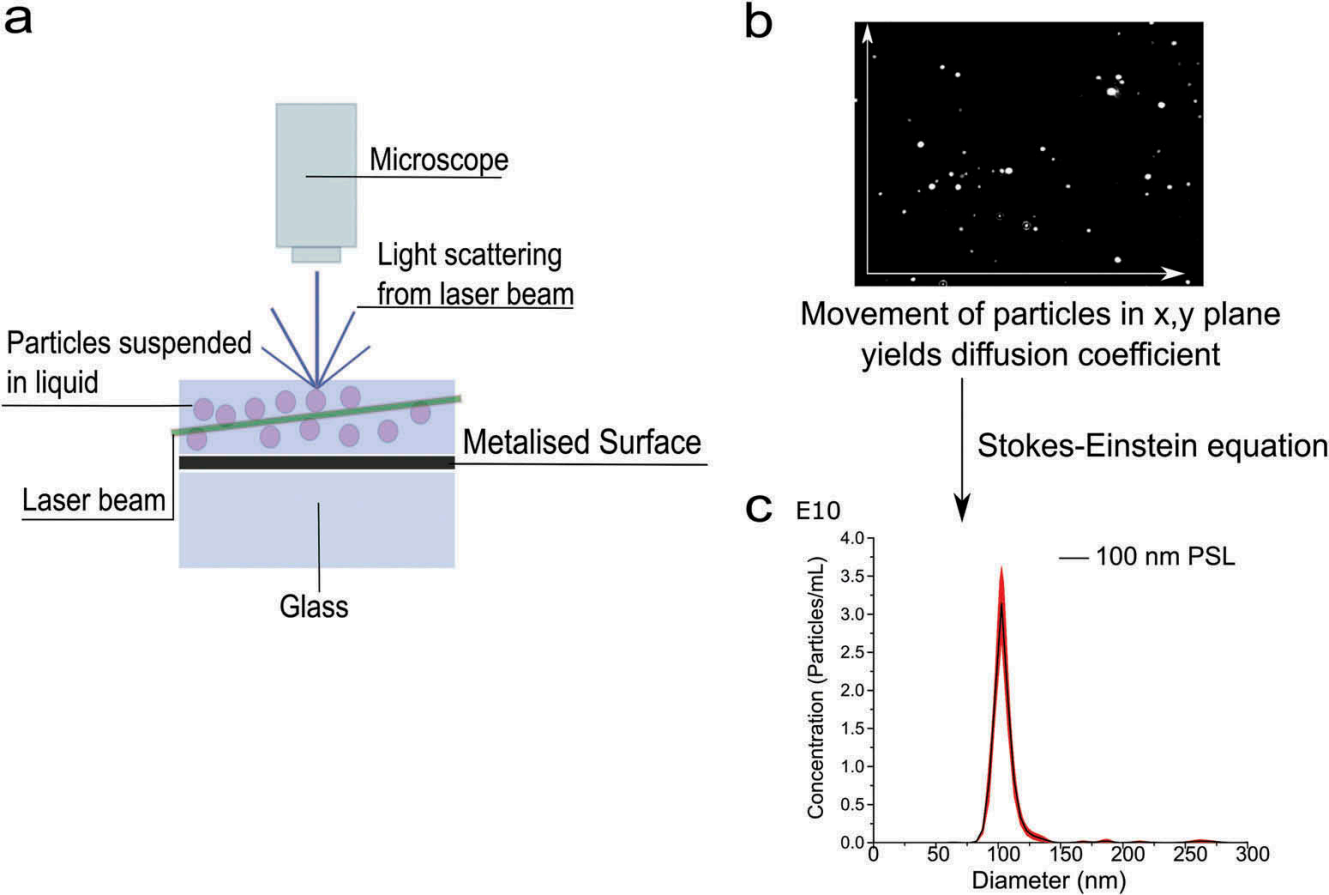
Schematic of a PTA set-up. Particles in suspension cause the scattering of light from a laser beam, with a microscope and camera detecting and recording videos of this scattered light (a). The PTA software then analyses the videos, allowing for the determination of the particle number, and the diffusion coefficient (b). The diffusion coefficient is then used to calculate the particle hydrodynamic diameter (c). Nanomaterials used for PTA measurement are commercial polystyrene latext (PSL) nanoparticles of 100 nm in diameter.

In the case of PTA, the software tracks individual particle movements to calculate the diffusion coefficient for each individual particle. It is a high-resolution analysis technique that is able to distinguish small differences between two particles or populations, either based on diffusion and Brownian motion or light scattering intensity. The software records a series of video files (of typically 30–60 s duration) of the particles viewed and then simultaneously identifies and tracks the centre of each particle on a frame-by-frame basis. The image analysis software then determines the average distance moved by each particle in the *x* and *y* directions. This value allows the particle diffusion coefficient (*D_t_*) to be determined and again using the Stokes–Einstein equation, the hydrodynamic diameter can be calculated, as demonstrated in Figure 2.

As Brownian motion occurs in three dimensions and PTA observes motion only in two dimensions, a variation of the equation must be used. It is possible to determine *D_t_* from measuring the mean squared displacement of a particle in one, two or three dimensions at a given time (*t*) (Equation 2(a,b,c), respectively) [18,19]

(2a) $$\bar{\left(x^{2}\right)}=\;\frac{2Tk_{B}t}{3\pi \eta d}$$

(2b) $$\bar{{\left(x,y\right)}^{2}}=\;\frac{4Tk_{B}t}{3\pi \eta d}$$

(2c) $$\bar{{\left(x,y,z\right)}^{2}}=\;\frac{2Tk_{B}t}{\pi \eta d}$$

Thus, Equation (2b) is employed when the particle movement is tracked in two dimensions.

One crucial advantage that the PTA over other measurement techniques is that it is not biased towards larger particles or aggregates. The software is based on the tracking of single particles, whereas typical DLS techniques place a strong bias on the largest particles present in the sample [17]. This allows for the detection of secondary peaks, which may not be possible with other traditional measurements. The counting of individual particles also allows to simultaneously measure the concentration of NPs, as the volume of the field of view is known. It should be noted however that the area of observation is limited to this field of view of the microscope and this can result in limitations to the number of particles tracked and the duration of the tracking. The introduction of new particles at regular intervals can help to overcome some of these issues by increasing the number of particle tracks, and ultimately the statistical robustness of the measurement.

## Breaking down PTA and NTA measurements

PTA is a higher-resolution size-measurement technique compared to DLS, and thus higher demands are placed on its precision and accuracy. The technique has a validated size limit between 30 and 600 nm [20], but may be pushed above and below these values depending on sample type, inherent light scattering potential and laser wavelength and camera sensitivity. It should be noted that this detection range is much narrower than DLS, which is between 0.7 nm and 10 µm. NTA has the ability to detect and analyse particles smaller than 30 nm by increasing the viscosity of the sample, for example [20]. This will lower the Brownian motion of the particles enabling improved tracking. Similarly, if the particle is a strong light scatterer, such as silver or gold, the detection limit for monomodal samples can be pushed lower again. The introduction of the Finite Track Length Adjustment (FTLA) algorithm has facilitated reproducible NTA size measurements, which are essential for comparability of particles sizes over time and space. Hence an in-depth understanding of all potential effects allows the user of the NTA techniques to control the measurement process so that highly reproducible results are common, and any outlier can be attributed to individual causes. The cause and effect analysis, first developed by Kaoru Ishikawa to improve the quality control in manufacturing [21], has also been applied to analytical chemistry and techniques [22] to obtain a comprehensive overview and understanding of all the effects onto a given measurements. While the diagram and parameters listed below are based on the NTA technique, many of the components are transferable to other PTA methods such as the Viewsizer 3000 (Manta Instruments Ltd., San Diego, CA, USA) and ZetaView (Particle Metrix GmbH,  Inning am Ammersee, Germany).

**Figure 3.**
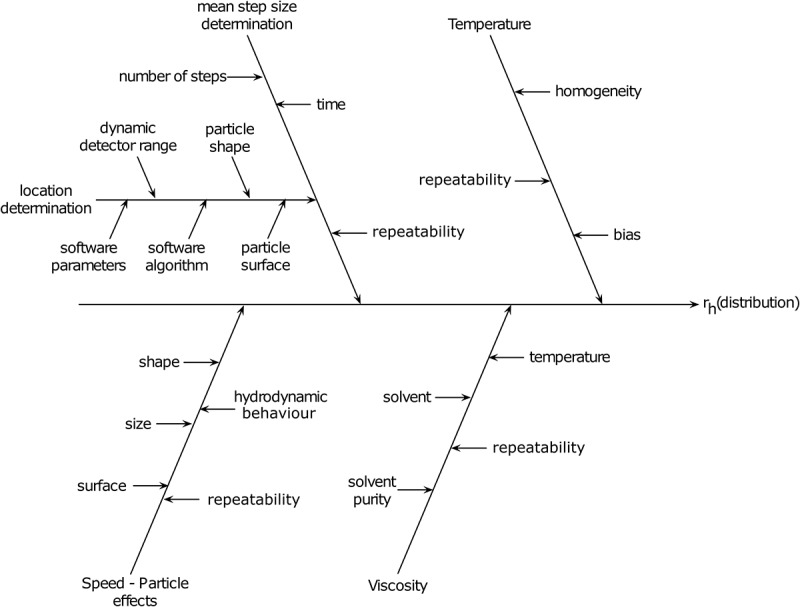
Cause and effect analysis of various parameters that will influence the measurement of a sample by NTA. The parameters that directly influence the calculation of the particle diameter include the mean step size determination, temperature, solvent viscosity and particle speed effects. Variations, or unknowns, in these factors will directly influence the particle hydrodynamic size distribution data (*r_h_*: hydrodynamic radius).

The procedure of the cause and effect analysis (Figure 3) starts with the equation of the measure, which is the slightly transformed Equation (2b). The main branches of the diagram are the variables of the equation, whereas natural constants, such as the Boltzmann constants (*k_B_*) or *π*, are omitted. They are the temperature (*T*), mean step size determination ($\bar{{\left(x,y\right)}^{2}}$), viscosity of the solvent (*η*) and the effects influencing the hydrodynamic behaviour of the particles.

### Temperature

According to the Brownian motion model, the square mean step size ($\bar{{\left(x,y\right)}^{2}}$) of a particle is proportional to the diffusion coefficient (*D*). As such, an increase of the temperature increases the squared mean travelled distance. Hence the homogeneity of the temperature field of the NTA measurement block, and especially of the observed region, is essential. In addition, slight differences in the temperature field can lead to convection currents, altering the movement and diffusion of the particles. Similarly, the temperature reading must be accurate (not biased) and repeatable, as it directly affects the measured size.

### Viscosity of the solvent (η)

The viscosity of the solvent has a direct effect on the Brownian motion of the particles, and, therefore on their perceived sizes. Increased viscosity solvents, relative to that of water, can impede the diffusion of the particles, making them appear slower, and ultimately resulting in a larger calculated size. In general, the viscosity of the solvent directly depends on the type of solvent being used, its purity and its temperature. Similarly, different solvents or solvent mixtures can affect the hydrodynamic diameter of the particles through van der Waals interactions and binding of solvent molecules to the particle.

### Diffusion coefficient: mean step size determination and its influencing factors

In PTA-based techniques, the diffusion coefficient, which is required to solve for the hydrodynamic diameter (Equation (2b)), is calculated by measuring the mean step size of the particle (the mean displacement of the particle over a given time).

In the case of NTA, the instrument generally records a video of the moving particles at a given frame rate, which allows for the determination of the location of all the particles in the observation area for each recorded frame. This allows the software to construct for the observed particles, the travelled tracks together with the number of steps, which it took to travel the distance.

The computer program analyses each frame of the video determining the location of the observed particles. As particles are not depicted as ideal round shapes, the computer program tries to identify the centre of the particles based on its recorded shape. This shape identification is influenced by the shape of the particle itself, the surface of the particle, which reflects the laser light into the detector, and by the dynamic range of the detector. The detector recording the position of the particle might get saturated depending on the focal plane of the microscope and the amount of the reflected light. This will cause a blur of the recorded particle shape and influence the determination of the centre of the particle. In contrast, not enough scattered light of a particle might mislead the particle centre determination. In general, only the highest reflecting parts of the particle surface are recorded, and this will vary rapidly due to the Brownian motion. Therefore, it proves essential to define an appropriate lower cut-off limited and at the same time ensure that the detector is adequately saturated. The former is done during the analysis of the recorded pictures, whereas the latter is adjusted before the recording of the Brownian motion with the setting of adequate detector sensitivity.

After analysing the position of the particles in a given frame, the computer program performs the additional task determining which particles form continuous tracks, which observed tracks have stopped and which particles appear new in the recorded area. The whole task might become challenging, when the number of particles in the recorded area exceeds certain limits. In such a case, too many collisions with energy transfer from one particle to another will affect the observation motion pattern. It will be challenging for the software to pick up all such energy transfers. On the other side, if the computer program stops each of the tracks after a collision then the tracks may be too short for determining a reliable mean step length.

It has been shown that PSDs determined from few-step tracks are excessively broadened. This is caused by the non-linear transformation of the steps length distribution to the PSD. FTLA permits to adjust this transformation according to the given number of steps, reducing the artificial broadening of the particles size distribution.

Other factors that influence the NP diffusion coefficient and hydrodynamic diameter include speed-particle effects. The hydrodynamic diameter describes the size of the particles and with it ‘an electrical double’ layer of solvent molecules. At least practically, they move with the particle themselves. This ‘bound’ layer depends on the interaction between the surface of the particles and the solvent molecules, where ions in solution first absorb onto the NP, with a second layer of ions associating to this layer via Coulomb forces. As such, the particle hydrodynamic diameter is heavily dependent on the solution that it is measured in. Hydrogen bonds and van der Waals forces influence the strength of the solvent molecule cloud, which moves along with the particles and defines their hydrodynamic diameter. These forces also play key roles in the properties of the material, enabling NP functionalisation for example. Thus, the size and especially shape of the particles, including their surface characteristics and functional groups, have additional effects onto the size and stickiness of the electrical double layer. Changes in NP properties that can influence such effects will influence the measured diffusion coefficient, and ultimately the particle hydrodynamic diameter.

## Differences between DLS and NTA measurements

**Figure 4.**
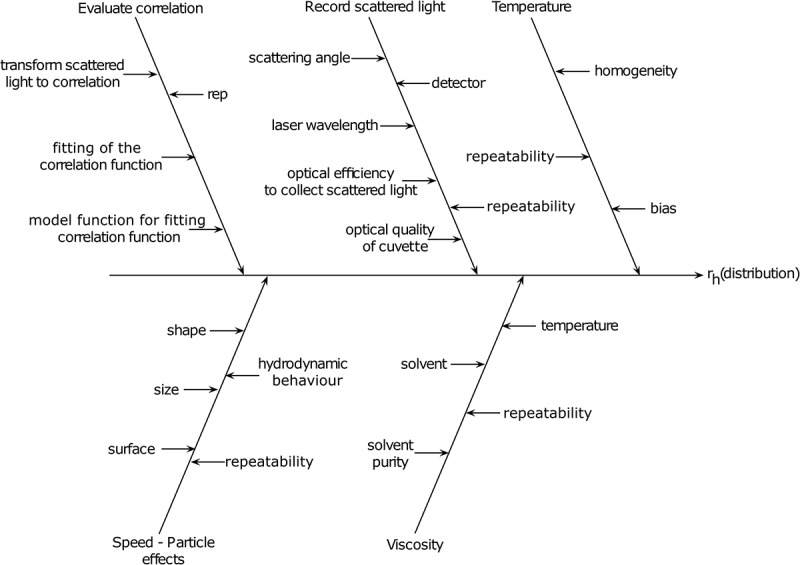
Cause and effect analysis of various parameters that will influence the measurement of a particle Z-average hydrodynamic diameter by DLS. The parameters that directly influence the calculation of the particle diameter include the creation of correlation function from the scattered light, as well as the parameters consistent with NTA such as temperature, solvent viscosity and particle speed effect. Variations, or unknowns, in these factors will directly influence the particle hydrodynamic size distribution data (rep: repeatability; *r_h_*: hydrodynamic radius).

The cause and effect analysis of DLS is similar to that of NTA, particularly in relation to temperature, speed-particle effects and solvent viscosity, as these are fundamental elements of the Stokes–Einstein equation (Equation 1). Where the two techniques differ, however, is in relation to the recording of the scattered light and the evaluation of the correlation function. In summary, these areas relate to the collection, and subsequent analysis of the scattered light to determine the particle hydrodynamic diameters. Figure 4 presents the evolution of Z-average particle diameter, with material refractive indices and absorption values also being required for more advanced analytical models. As shown, light scattering intensity fluctuations from the nanomaterials are collected and assessed, and subsequently used to generate the intensity-based light scattering size distributions. Further computational processing is then required to transform this data to number, and then volume-based distributions. This could then lead to uncertainties and approximations in the data fitting. A number of comparative studies comparing DLS and NTA measurements are available [17,23], each with different conclusions about the applicability of the methods. However, it should be noted that the progress in software and data-processing improvements in recent years, along with refinement of the instrumentations themselves, has enhanced the resolving capabilities and limits of detections of both techniques. As such, the currently available literature may not accurately reflect the current state-of-the-art technologies and improvements across all instrumentations.

## Advanced characterisation of polydispersed samples

**Figure 5.**
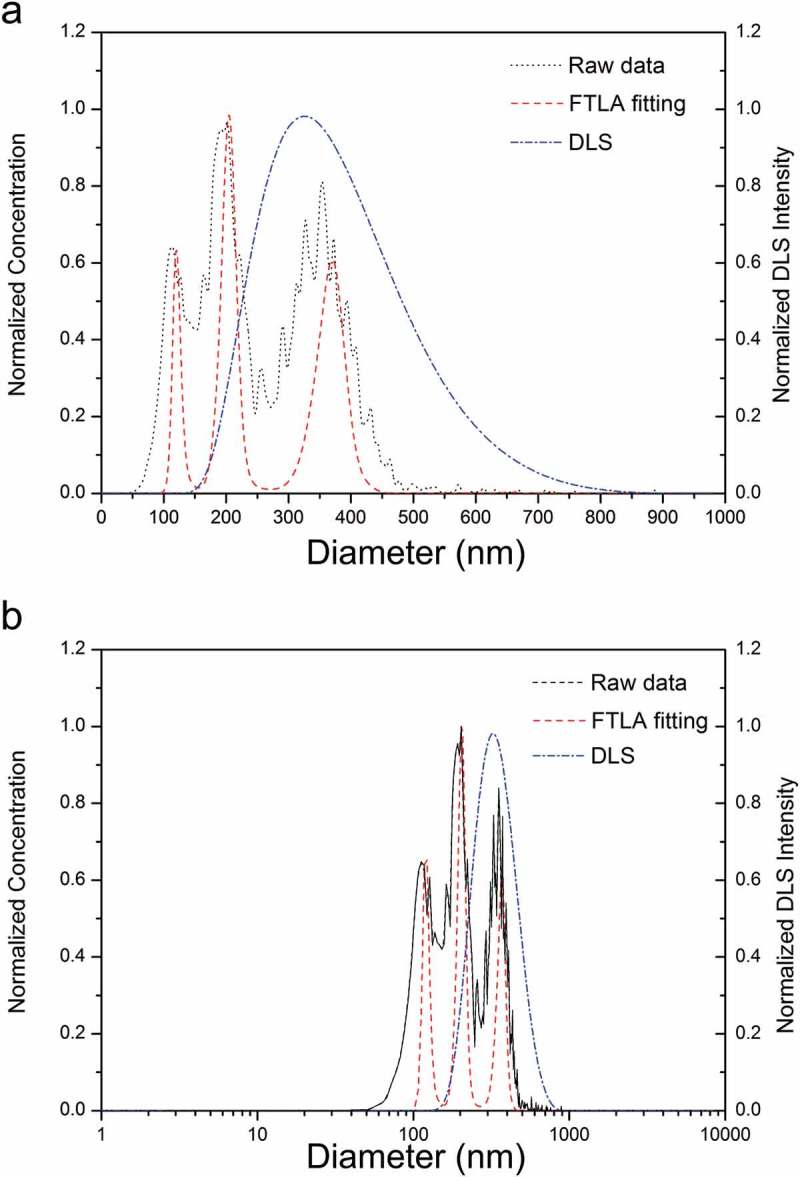
Analysis of a 100, 200 and 380 nm PSL NP mixture using NTA and DLS. Black dotted line represents raw unprocessed NTA data, red dashed line represents NTA data following FTLA being applied, and blue line represents the distribution of the same sample as analysed by DLS. (a) Transformation of the DLS data from a log to linear scale, (b) original data.

DLS is presently one of the most common methods used in NP sizing; it has its limitations which have an impact on the characterisation of polydispersed samples. It is an ensemble-based, intensity weighed methodology, which is generally only suited for mono-dispersed samples. Conversely, this is not the case for PTA, as it is not influenced by the presence of larger particles. Furthermore, relating specifically to NTA, the incorporation of FTLA has advanced this by improving size distribution peak isolation and resolution [24,25]. The FTLA algorithm accounts for the tracking of a particle over a finite number of frames leading to a statistical error in the average particle diameter [24]. Where a polydispersed sample is analysed using DLS, the Z-average or cumulants mean will consist of only one value, weighted towards the largest component, as demonstrated in Figure 5. The polydispersity index (PDI) can be used to inform the user of the degree of polydispersity in the sample, and is generated based on the width of a hypothetical Gaussian distribution. A PDI greater than 0.4 indicates that the sample is polydisperse. The relative standard deviation (RSD) can be calculated based on NTA data, and acts in a similar manner, with a RSD greater than 40 indicating a polydispersed sample.

In more general terms, NTA has a much greater resolving power than to DLS. Less than a 50% difference in size is needed for peak resolution by NTA, while DLS requires a greater than three-fold difference in size [17]. The superior resolving capabilities offered by the PTA method aid in the synthesis and manufacturing of NPs by enabling faster in-line or batch-mode monitoring of key particle properties. It should be noted however that DLS has a lower limit of detection, compared to PTA-based methods, which enables the characterisation of NPs present in lower tens of nanometre size range.

One crucial difference between DLS and other techniques is that DLS distributions are typically a symmetrical, normal (Gaussian) function plotted in a log scale. Upon transforming the log scale distribution into a linear scale, a tailed distribution will be derived, as shown in Figure 5(a) (dashed and dotted blue line). As a result, comparing the size distributions of a sample, using multiple techniques, can be challenging and inaccurate.

As previously stated, DLS is an average-based technique, with the Z-average diameter representing the light scattering intensity average of the sample. In contrast, single-particle analysis techniques, such as PTA, or single-particle inductively coupled plasma mass spectrometry or quantitative-TEM accurately measure individual particle. These single particle, or analyte, techniques are becoming increasingly utilised in a number of other analytical areas, with techniques such as single-cell mRNA sequencing operating under a similar principle, compared to the ensemble measurements via bulk RNA sequencing [26,27], since they allow for the analysis of the true variability within a sample or population.

While more advanced and technically demanding techniques such as asymmetrical flow-field-flow-fractionation DLS (AF4-DLS) can resolve complex and polydispersed samples, several other light scattering-based batch-mode techniques also exist to characterise such samples. These include the VASCO Flex^TM^ Particle Size Analyzer (Cordouan Technologies, Pessac, France) and the ANALYSETTE® 12 DynaSizer (Fritsch GmbH, Idar-Oberstein, Germany), which have the ability to calculate size distributions of complex or polydispersed samples using the Padé-Laplace and Sparse Bayesian Learning (SBL) algorithms. As such, both instruments can be used in the kinetic monitoring of changes in particle sizes.

**Figure 6.**
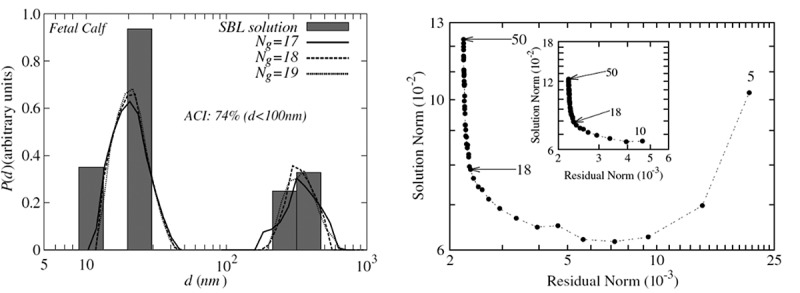
The use of Sparse Bayesian Learning (SBL) to reconstruct bimodal size distributions from DLS data (a). These data are obtained from the L-curve of the reconstruction. A value of Ng = 18 yields the most probable particle size distribution (PSD), as it is the lowest value on both *x*- and *y*-axis (b). Reused with permission from Nyeo and Ansari [28].

Due to the discrete distribution of the Padé-Laplace algorithm, it is not possible to obtain size-distribution plots for the components of a polydispersed sample. This can be overcome by using the SBL analysis modes to generate size distributions for the sample [28]. Here, the size distribution is calculated based on the residues (quality of fit) (*x*-axis) and sparsity index (size of the regularised solution (*Ng*)) (*y*-axis) of the L curve [28], with the most probable PSD having the lowest sparsity and residue index. Briefly, this L curve is a visualisation of the trade-off between the complexity of the fitting, i.e. the number of exponentials and corresponding size of the solution (*Ng*) and the quality of the fitted line. The optimal *Ng* lies at the vertex of the L curve. True distribution in the data can be difficult to obtain in this manner; however, as noise in the data can limit the detection of components of the system [28]. The work by Nyeo and Ansari [28] demonstrates the use of SBL to reconstruct bimodal distributions from DLS data, as shown in Figure 6. Here, the authors use SBL to reconstruct the PSDs of crystalline protein from the ocular lens using experimental DLS data.

One other key benefit of the VASCO flex system is the ‘*in situ* probe’. This allows the user to measure the size distribution of their sample *in situ*, thereby allowing for the measurement of particle size and stability during the course of their synthesis, for example. This can be a very powerful and beneficial for material scientists, allowing them to monitor their reactions in real-time, in an environment that may not typically allow for continuous assessment. In the case of nano-pharmaceuticals, the ability to maintain the sterility of the formulation is of paramount importance, and thus this system can provide scientists the ability to characterise their sample directly in the reaction vessel.

Coupling of the VASCO flex system and the SBL algorithm with AF4 has also allowed users to identify the size distributions present within a bulk and fractionated polydispersed nanoplastic mixtures and fullerene aggregates [29,30]. Here, the algorithm allowed for the identification of two distinct populations in the different formulations, in a manner similar to the use of standard DLS instruments without the added resolving capabilities.

It should be noted, however, that the analytical models discussed above can ultimately obscure the true data in the distribution. Increasing the number of parameters in the correlation function can improve the curve fitting of the data. However, this improved fitting does not necessarily mean that it represents the true distribution of the data.

**Figure 7.**
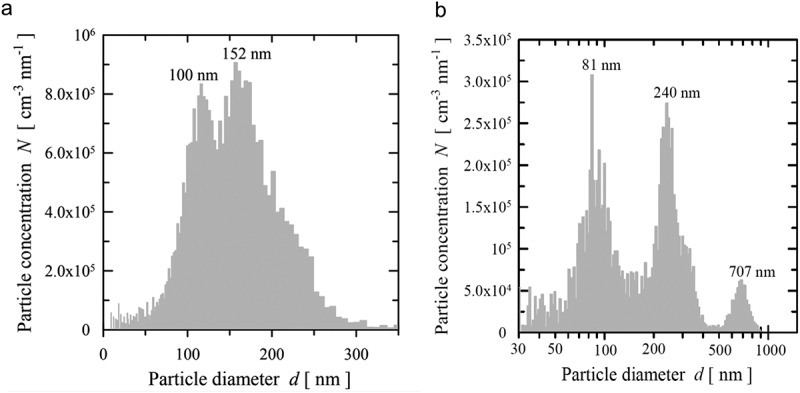
Representative graphical output for the analysis of a bimodal (a) and trimodal sample (b) using the Viewsizer 3000 system. Like NTA systems, the instrument can resolve two particle populations with 50% different average sizes. The ability to resolve the 707 nm (nominal) component of the trimodal mixture would be difficult for NTA due to the light scattering obscuring the smaller components of the sample. Reused with permission from MANTA Instruments [31].

While the visualisation of particle light scattering is a distinct advantage for PTA over other techniques, the dynamic range of the camera and the laser set-up can also introduce the bias and uncertainties. Under Rayleigh and Mie scattering, a shorter wavelength light source is required to visualise smaller NPs due to their light scattering potential. The use of a short wavelength laser in NTA set-ups, such as the 405 nm laser, can allow for the detection of the smaller components of a polydispersed sample. However, this can cause the larger particles to blind the detector, leading to the small particles becoming obscured. The Viewsizer 3000 system overcomes some of these issues by incorporating three lasers and a colour camera to observe the Brownian motion of the particles under each wavelength [31]. The power of each laser can be tuned for each component of the polydispersed sample, to maximise the particles when tracked and analysed, as demonstrated in Figure 7. This theoretically makes the Viewsizer 3000 superior to NTA; however, greater study and validation of the system is required, since recently launched on the market.

## Concentration measurements

The measurement of particle concentrations is a requirement across a range of applications, from the synthesis of engineered particles to the characterisation of NPs of biological origin. Accurate concentrations are required when studying the effect of a vaccine on virus titres, or where the concentration of exosomes and nano-vesicles may be correlated to disease states, for example. NTA provides a mean to determine these particle concentrations in a reliable and reproducible fashion, while simultaneously measuring particle sizes [25]. It has been demonstrated that the concentration upgrade (an instrument-specific software correction) for the NTA system can lower the measurement uncertainty of a sample from 170% to 10%, with the upgrade giving a linear response over a range from 8.6 × 10^6^ to 5.7 × 10^9^ particles per millilitre [25].

The light scattering potential of the particles again plays a role in accurate concentration measurements, as it does with size determination. Particles with low refractive indices will inherently result in more uncertain results due to the systems operation limits. While the concentration measurement ability of the Viewsizer 3000 has not been fully investigated, as stated above, the ability to use multiple lasers set-ups has the potential to allow the system to surpass NTA in this area.

**Figure 8.**
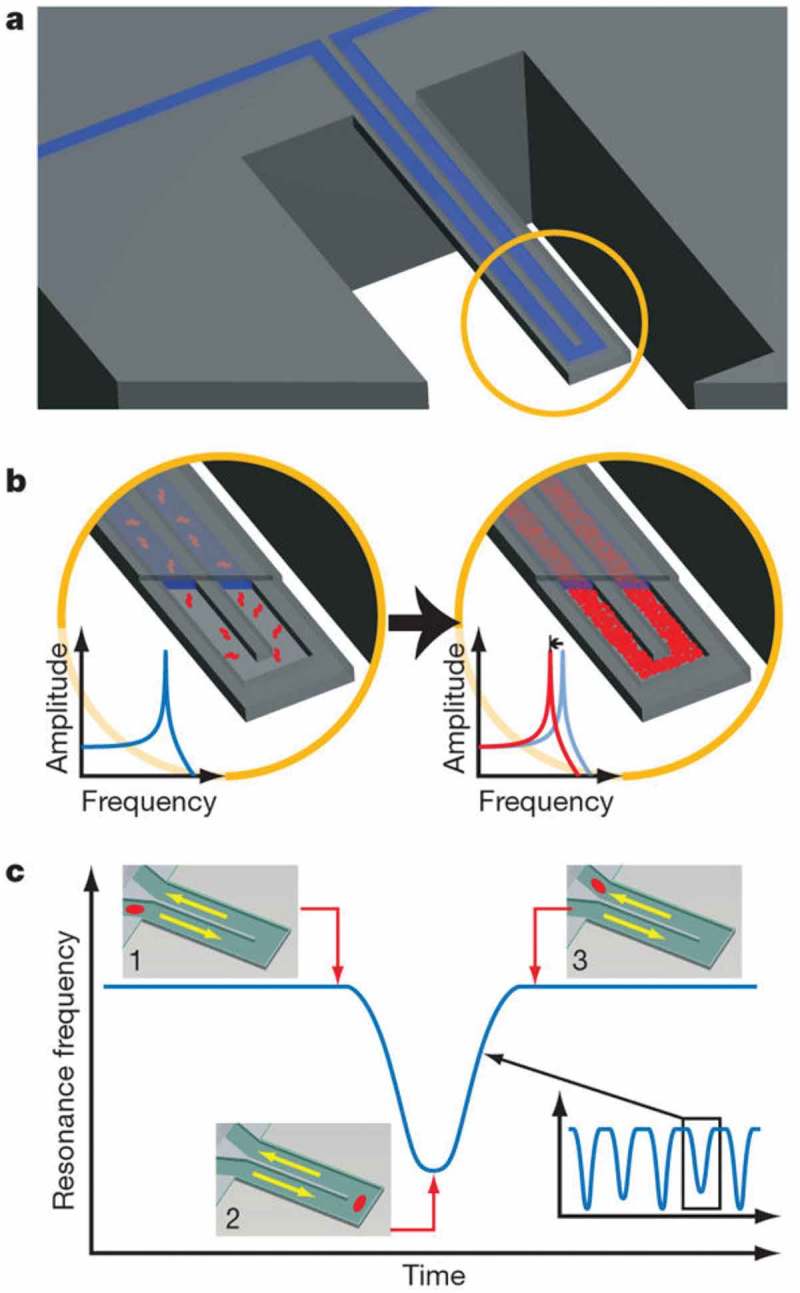
Schematic overview of resonant mass measurement, using systems such as Archimedes (Malvern Panalytical Ltd.). A suspended microchannel translates mass changes into changes in resonance frequency. Fluid continuously flows through the channel and delivers biomolecules, cells or synthetic particles (a). While bound and unbound molecules both increase the mass of the channel, species that bind to the channel wall accumulate inside the device, and, as a result, their number can greatly exceed the number of free molecules in solution. This enables specific detection by way of immobilised receptors (b). In another measurement mode, particles flow through the cantilever without binding to the surface, and the observed signal depends on the position of particles along the channel (insets 1–3). The exact mass excess of a particle can be quantified by the peak frequency shift induced at the apex (c). Reused with permission from Burg et al. [32].

The Archimedes system (Malvern Instruments Ltd., Malvern, UK) also has the ability to simultaneously determine particle size and concentration in solution. However, unlike PTA systems, Archimedes operates under the principle of resonant buoyant mass, rather than light scattering. The resonating frequency of the cantilever changes as particles of different buoyant mass pass through the flow chamber, as demonstrated in Figure 8.

These frequency changes can then be used to calculate the dry mass and size of each particle, once the density of the material is known. This technique has been used to weigh single NPs, single bacterial cells and sub-monolayers of adsorbed proteins in water with sub-femtogram resolution (1 Hz bandwidth) [32]. In terms of pharmaceutical applications, the ability of the technique to characterise samples based on their buoyant mass allows users to distinguish between engineered nanomedicine products or protein aggregates, and silicon oil in syringes, for example. Applications such of this are of interest to material scientists and regulators as larger protein aggregated or indeed oil from pre-filled syringes may pose immunological risks. However, as with all techniques, there are limitations, with different resonator set-ups required in order to analyse different sized samples, and ultimately there are minimum detection limits based on the material being analysed.

Other non-PTA-based systems that can measure particle size and concentration include Zetasizer Ultra (Malvern Panalytical, Malvern, UK) and resistive pulse sensing (RPS) variations, such as tunable-RPS (TRPS; Izon Sciences Ltd., Christchurch, New Zealand) and nCS1 (Spectradyne LLC, Torrance, CA, USA). Zetasizer Ultra is a relatively new instrument on the market, and its capabilities in measuring particle concentration are yet to be fully tested. Single-particle inductively coupled plasma mass spectrometry can also be used to calculate particle sizes and concentrations, but is generally suited only for metal-containing NPs. To counteract this, analytical centrifugation can be utilised to determine PSDs and concentrations [12,33]. This technique does not require the use of a calibrator as it is a first-principles-based method that separates and characterises materials based on their density. As such it is not substance specific, and can be used to characterise enumerate materials [12]. Another promising technique for measuring particle concentration is laser-induced breakdown detection (LIBD). Here, particle concentration can be correlated to the energy curves based on the formation of individual plasma events produced when pulsed, focused laser beams collide with a NP [34,35]. Further work is required to fully validate the technique; however, as dispersion agents in a sample can dramatically affect the recorded particle concentrations [34], possibly limiting its applications. The concentration of the sample is calculated based on a calibration curve generated from a known standard, and is an ensemble method. However, the technique can detect and analyse samples with very low concentrations which may not be detectable by light scattering methods [35]. Small-angle X-ray scattering (SAXS) can also be used to determine NP sizes and concentrations [36–38].

However, it should also be noted that due to the difficulty in obtaining certified reference materials (CRMs) for concentration measurements, the ability to generate a calibration curves for LIBD and SAXS of appropriate quality may prove to be challenging. The lack of CRMs for concentration measurements also has implications across all concentration measurement techniques, where if the traceability of a measurement is not documented, it is not possible to comment on the accuracy of the method. Hence, it only allows for the determination of the precision and repeatability of the measurement. A number of research institutes, including the US National Institute of Standards and Technology (NIST) and the National Physical Laboratory, are currently working to address these problems.

### Characterisation of NPs of biological origin

As stated above, there are a number of benefits for each particle characterisation system, and the applications that these benefits lend themselves to. In the case of the characterisation of NPs of biological origin, the NTA technique offers the greatest flexibility and robustness [17,39,40]. The ability to analyse samples in simple and complex biological media [3,19,25,41,42] is of distinct advantage when characterising materials under physiological conditions. Size distribution analysis can be accurately and robustly performed for each of the populations in the sample.

**Figure 9.**
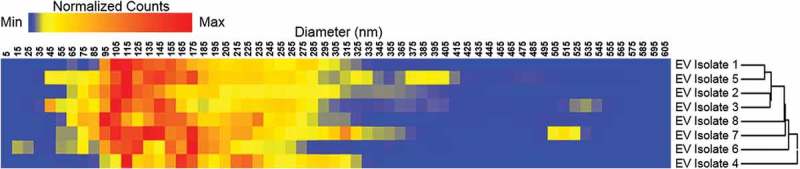
Heat map of scale-normalised (0–1,000,000) nanoparticle tracking analysis profiles (5–605 nm) depicting relative quantities of particles by size and their relationship based on hierarchical clustering. Data represent average readout from six nanoparticle tracking analysis measurements. EV: extracellular vesicle. Each line of the heat map is a representation of NTA size distribution for that particular nano-vesicle, with the colour gradient reflecting the lowest (blue) to highest (red) particle concentration for a given size range.

High-resolution analysis of the sample can be used to identify small differences between the components. For examples, NTA can be used to identify variations in nano-vesicle sizes that arise due to the method of isolation. Gerlach et al. demonstrate that different lectins used to isolate nano-vesicles from urine resulted in vesicles of varying sizes [13]. A number of visualisation methods can be used to illustrate these variations in size and their relationships with other vesicles. The relative size and concentration ranges of the isolated vesicles are presented below as a heat map, as shown in Figure 9. When comparing large data sets, these visualisations allow for the improved identification of subtle variations between groups. Further biochemical or proteomic analysis of the samples, along with the size and concentration data, can allow users to cluster or group subsets of vesicles into groups of interest, or biological importance.

**Figure 10.**
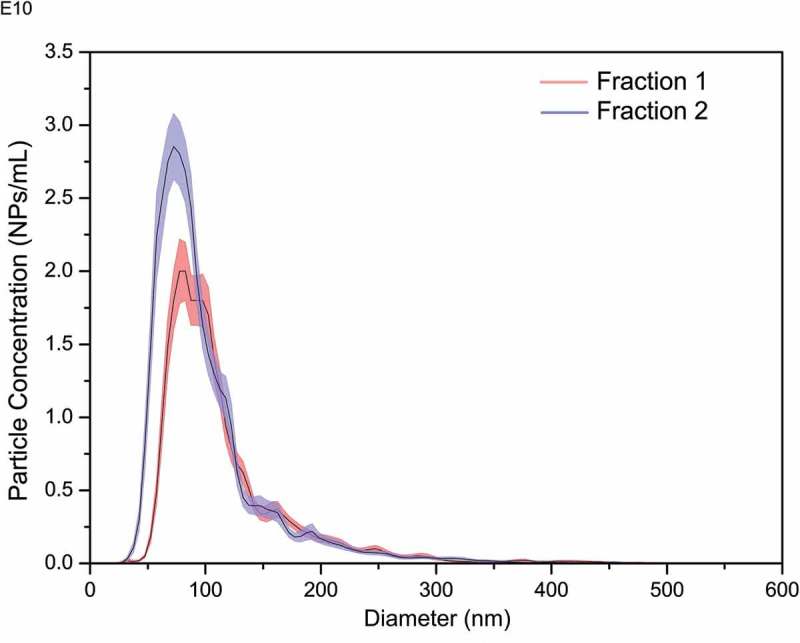
Size distribution plots showing differences in size and concentration for two fractions of extracellular vesicles isolated using qEV isolation columns (Izon Science Ltd., UK). Blue and red shading reflect ±standard deviation of 6 NTA measurements (solid black line). Fraction 1: mode size 72 ± 2 nm; Fraction 2: mode size 87 ± 2 nm (LBCAM (TCD) unpublished data).

Similarly, NTA has also been used to show differences in the size distributions of micro-vesicles isolated from different cell lines [42], and the difference in the size distributions of extracellular vesicles isolated using size exclusion chromatography (Figure 10). These high-resolution capabilities can open up new avenues for use of PTA method in diagnostic and clinical applications where differences in vesicle sizes, distribution widths or concentrations may be prognostic or clinical indicators, for example.

PTA can visualise and characterise fluorescent NPs allowing for differentiation between circulating protein aggregates and a biological NP of interest [43].

Again, it should be noted that PTA-based methods are not the only technique suited to the characterisation of biological samples. TRPS-based systems, such as qNano Gold, can measure the size and concentration of such samples, particularly viruses and extracellular vesicles. It has been demonstrated that size exclusion chromatography and TRPS can be combined to monitor the production and purification of viral particles, for example [44].

As mentioned above, samples with broad PSDs, particularly those of biological origins, can be difficult to analyse. For this reason, care should be taken when choosing a measurement technique [45]. In cases of complex multimodal samples, TRPS can have a greater resolving power than PTA, where it is able to analyse all particles present in the sample [45].

The high-resolution capabilities of PTA- and RPS-based instruments may not be entirely relevant in all applications; however, as the given uncertainty in the measurement may be outside of the range for biological responses. Statistically significant changes in virus or exosome size or concentrations may be detected by the technique, but these values may still lie within a physiological, clinical, or therapeutic range. For this reason, the level of uncertainty required, the expected particle size range, and the nature of the responses should be determined prior to analysis [46].

## Conclusions

We presented a number of characterisation techniques which can determine NP size and concentration in liquid suspension, each with their own inherent advantages and disadvantages. A summary of the instruments discussed in this review is presented in Table 1.

**Table 1.** Parameters of nanoparticle sizing and concentration measurement instruments listed in this review.

Instrument	Manufacturer	Measurement principle	Detection Limits^a^	Concentration measurement	Notes
Zetasizer Nano Series	Malvern Panalytical	DLS	0.3 nm–10 µm	No^b^	Gold standard particle analyser. Can be used in batch or flow mode. ^b^The new Zetasizer Ultra has the capabilities to measure both particle concentration and size, but has yet to be fully tested.
VASCO Flex	Cordouan Technologies	DLS	0.5 nm–10 µm	No	Relatively new system, with advanced data processing algorithms included. *In situ* probe allows for measurements inside of reaction vessels.
ANALYSETTE 12 DynaSizer	FRITSCH	DLS	1 nm–6 µm	No	Small (50 µL) sample volumes. Allows for analysis of thin layers.
NanoSight Series	Malvern Panalytical	PTA	10 nm–2 µm (10^6–^10^9^ particles/mL)	Yes	ASTM and ISO certified for particle size and concentration measurements. Single-particle analysis.
Viewsizer 3000	MANTA Instruments	PTA	10 nm–2 µm	Yes	A three laser set-up, with ability to tune the intensity of each laser to improve particle tracking. Relatively new with unproven capabilities
Archimedes	Malvern Panalytical	Resonant buoyant mass	50 nm–5 µm	Yes	Capable of providing information on sample concentration, viscosity, polydispersity, density and volume, and distinguishing between negatively buoyant proteinaceous particles and positively buoyant contaminating silicone oil droplets. Resonator must be changed depending on particle size
qNano Gold	Izon Science	TRPS	40 nm–10 µm	Yes	TRPS measures nanoparticles suspended in electrolytes on a particle-by-particle basis as they pass through a nanopore. The transient current pulse caused by a particle traversing the pore is directly proportional to particle volume, enabling a highly precise and repeatable measurement of size. Measured particles are compared to a NIST traceable calibration standard.
nCS1	Spectradyne LLC	RPS	50 nm–10 µm	Yes	The instrument, using only electronic sensing with no optical elements, rapidly counts and sizes individual nanoparticles in a sample, achieving few-per cent precision in both size and concentration. Instrument measures the individual diameters of each particle, so the particle size histograms provide quantitative, high-resolution measurements of both particle diameter and absolute concentration. Uses disposable microfluidic cartridges.
MAGELLAN	Cordouan Technologies	LIBD	10 nm–1 µm (10^4^–10^11^ particles/mL)	Yes	Ensemble method for calculation of particle size and concentration in low concentration samples. Used in characterisation of colloids in aquatic systems and trace analysis.

^a^Detection limits depend on material. Data sourced from manufacturers web pages; ^b^Concentration measurements are only available on Zetasizer Ultra configurations.

DLS = dynamic light scattering; PTA = particle tracking analysis; TRPS = tunable resistive pulse sensing; RPS = resistive pulse sensing; LIBD = laser-induced breakdown detection.

We identify a number of key parameters that can influence the uncertainty in DLS and PTA measurements, and outline techniques and processes that allow a user to gain a better understanding of their particles under a given set of conditions. The presented examples of the measurements of particle sizes in suspensions will help scientists understand their results and comply with regulatory demands for accurate and reliable particle size and concentration data.

To select the most appropriate characterisation techniques, the scope of the measurement and the uncertainty level must be considered when generating solid data in support of best scientific practice for nanomaterial-containing product development.
